# Supplementary material for: Factors influencing the use of emergency contraceptives among reproductive age women in the Kwadaso Municipality, Ghana
Source: PLoS One. 2022 Mar 3;17(3):e0264619. doi: 10.1371/journal.pone.0264619 (PMC8893659; doi:10.1371/journal.pone.0264619)
Supplement: S1 File — (DOCX) [file pone.0264619.s002.docx]

**APPENDIX I: DATA COLLECTION TOOL**

**QUESTIONNNAIRE**

SCHOOL OF PUBLIC HEALTH

COLLEGE OF HEALTH SCIENCES

KWAME NKRUMAH UNIVERSITY FOR SCIENCE AND TECHNOLOGY KUMASI

**Study Title**: Factors influencing the Use of Emergency Contraception among Reproduction Age Women in the Kwadaso Municipality, Ghana.

| **Section A: Background Information** | | |
| --- | --- | --- |
|  | ID …………………………. | EC[__\|__\|__] |
| 1 | Date of interview (dd/mm/yyyy) | [__\|__]/ [__\|__]/[__\|__][__\|__] |
|  | Name of interviewer ……………………………………… | Code  [__\|__] |
| 2 | Name of Sub-municipality ………………………………………….. | Code  [__\|__] |

| **Section B: Socio-demographic and Economic Characteristics** | | | |
| --- | --- | --- | --- |
| Q. No. | | Questions and responses | Response code |
| 1 | | How old are you? (**reference to last birthday**)  (Write exact age in years) ………………………. | [__\|__] |
| 2 | | What is your Religion?  01=Christianity \| 02=Islam \| 03=Traditional  04=Other (specify)…………………………………. | [__\|__] |
| 3 | | What is your highest educational level?  01= No formal education  02= primary  03= Some High School  04= Completed High School  05= Vocational degree or certificate (e.g. electrician’s license, auto repair certicate)  06= Bachelor’s degree (college or university undergraduate, BA, BS, BArch, BEng, etc.)  07= Graduate or advanced professional degree (MBA, PhD, JD, MD, etc.) | [__\|__] |
| 4 | | What is your current marital status?  01 = Married \| 02 = Single \| 03 = Divorced  04 = Separated \| 05= Widowed \| 06 = Co-habitation | [__\|__] |
| 5 | | If married, what is the highest educational level of your partner?  01= No formal education  02= primary  03= Some High School  04= Completed High School  05= Vocational degree or certificate (e.g. electrician’s license, auto repair certicate)  06= Bachelor’s degree (college or university undergraduate, BA, BS, BArch, BEng, etc.)  07= Graduate or advanced professional degree (MBA, PhD, JD, MD, etc.) | [__\|__] |
| 6 | | Dou you do any type of work for pay in the last month?  01= Yes \| 02 = No | [__\|__] |
| 7 | | If you did not work in the last month, what was the main reason you did not work?  01= No work available \| 02 = Seasonal inactivity  03= Student \| 04= Household / family duties  05= Too young to work \| 06= Infirmity / sickness  07= Other, specify …………………………. | [__\|__] |
| 8 | | How were you paid for your work (If currently unemployed use your last job)?  01= Regular wages or salary  02= Payment in kind  03= Casual labor (hourly/daily)  04= Unpaid contribution worker  05= Self-employed or own my business | [__\|__] |
| 9 | | Monthly Income (Gross personal income per month, including allowances, bonuses and other benefits, before any deductions/expenditure without savings?)  01= GHC 500  02= More than GHC 500, GHC 1,000  03= More than GHC 1,000, GHC 1,500  04= More than GHC 1,500, GHC 2,000  05= More than GHC 2,000  06= Don’t know  07= Refused | [__\|__] |
|  | | | |
| **Section C: Knowledge and Awareness of Emergency Contraception (EC)** | | | |
| 10 | Are you familiar with family planning or planned parenthood?  01= Yes \| 02 = No | | [__\|__] |
| 11 | Have you ever heard of Emergency Contraception?  01= Yes \| 02 = No | | [__\|__] |
| 12 | If yes, which was / were the source(s) of information? (**tick all that apply)**  01= Family \| 02 = Partner / boyfriend  03= Friend \| 04= Health professional  05= Church \| 06= Mosque  07= Media | | [__\|__]  [__\|__]  [__\|__]  [__\|__]  [__\|__]  [__\|__] |
| 13 | Are you aware of the risks associated with unprotected sex?  01= Yes \| 02 = No | | [__\|__] |
| 14 | What are some of the risks? (**tick all that apply**)  01= Unwanted pregnancies  02 = Contracting HIV/AIDS  03= Contracting other STIs (e.g. gonorrhoea, syphilis, etc.)  04= Other(s) Specify…………………. | | [__\|__]  [__\|__]  [__\|__]  [__\|__] |
| 15 | Do you know that ECs can be used to prevent unwanted pregnancy after unprotected sex?  01 = Yes \| 02 = No | | [__\|__] |
| 16 | Which of the following ECs have you heard of? (**tick all that apply**)  01= Combined oral contraceptive pills  02= Progesterone only pill. (e.g. Lydia, Postinor 2, etc.)  03= Intrauterine copper device  04= Ulipristal acetate  99= Don’t know | | [__\|__]  [__\|__]  [__\|__]  [__\|__]  [__\|__] |
| 17 | Is EC recommended as a regular contraceptive method?  01 = Yes \| 02 = No \| 99= Don’t know | | [__\|__] |
| 18 | What is the time limit for taking emergency contraceptive pills after unprotected sex?  01= Within 12 hrs \| 02= Within 24 hrs  03= Within 48 hrs (2 days) \| 04= Within 72 hrs (3 days)  99= Don’t know | | [__\|__] |
| 19 | Apart from EC do you know any other modern contraceptive method?  01 = Yes \| 02 = No | | [__\|__] |
| 20 | If yes, which of the modern methods do you know? (**tick all that apply)**  01= Injectable Depo provera  02= Implants (e.g. Jadelle)  03= Combined oral contraceptive pill (e.g. Secure)  04= Mini pills (i.e. progesterone only pills)  05= Condom  06= Intrauterine devices (IUD)  99 = Don’t know | | [__\|__]  [__\|__]  [__\|__]  [__\|__]  [__\|__]  [__\|__]  [__\|__]  [__\|__] |
| 21 | Where can emergency contraceptives be obtained? **(tick all that apply**)  01= Hospital / health centre  02= Community health worker  03= Private clinic  04= Pharmacy  05= Supermarket  06= Other, specify ……………….  99= Don’t know | | [__\|__]  [__\|__]  [__\|__]  [__\|__]  [__\|__]  [__\|__]  [__\|__]  [__\|__] |
| 22 | Are there side effects associated with the use of EC?  01= Yes \| 02= No | | [__\|__] |
| 23 | If yes, what are they?  …………………………………………………. | | [__\|__] |
| **Section D: Use of Emergency Contraception** | | | |
| 24 | Have you ever used any form of Emergency contraception?  01 = Yes \| 02 = No | | [__\|__] |
| 25 | If yes, did you use EC during your last coital activity?  01 = Yes \| 02 = No | | [__\|__] |
| 26 | If yes, when did you use EC during your last coital activity?  ……………………………………… | | [__\|__] |
| 27 | Did you use EC during your last but one coital activity?  01 = Yes \| 02 = No | | [__\|__] |
| 28 | If yes, when did you use EC during your last but one coital activity?  ……………………………………… | | [__\|__] |
| 29 | Did you use EC during your last but two coital activities?  01 = Yes \| 02 = No | | [__\|__] |
| 30 | If yes, when did you use EC during your last but two coital activities?  ……………………………………… | | [__\|__] |
| 31 | Why did you use emergency contraceptive? **(If yes to Q24, 25 or 26)**  01= Because I experienced condom breakage or slippage  02= Because I experienced failed coitus interruptus (withdrawal) during sex  03= Because of miscalculation of rhythm method  04= Because I had unexpected unprotected sex  05= Other, Specify …………………………………………………. | | [__\|__]  [__\|__]  [__\|__]  [__\|__]  [__\|__] |
| 32 | When did you use ECs to effectively prevent pregnancy after sex?  01= Within 24 hrs \| 02= Within 72 hrs  03= Within 120 hrs \| 04= Until menstrual period  05= After I missed a period \| 06= I don’t know | | [__\|__] |
| 33 | Where did you access ECs? **(*tick all that apply)***  01= Friends  02= Parents  03= Other family members  04= At a local clinic, with prescription  05= At a local clinic, without a prescription  06= At the pharmacy, over the counter  07= At the pharmacy, prescription only | | [__\|__]  [__\|__]  [__\|__]  [__\|__]  [__\|__]  [__\|__]  [__\|__] |
| 34 | Apart from ECs, which other modern contraception do you use? (tick all that apply)  01= Injectable Depo provera  02= Implants (e.g. Jadelle)  03= Combined oral contraceptive pill (e.g. Secure)  04= Mini pills (i.e. progesterone only pills)  05= Condom  06= Intrauterine devices (IUD)  07= Other, Specify…………………. | | [__\|__]  [__\|__]  [__\|__]  [__\|__]  [__\|__]  [__\|__]  [__\|__] |
| **Section E: Attitude towards Emergency contraception Use** | | | |
| 35 | The provision of EC to women would encourage promiscuity hence increase the prevalence of HIV/AIDS and other STIs  01= Strongly Agree \| 02= Agree \| 03 = Neutral  04= Disagree \| 05= Strongly Disagree | | [__\|__] |
| 36 | The provision of EC would discourage compliance to other contraceptive methods?  01= Strongly Agree \| 02= Agree \| 03 = Neutral  04= Disagree \| 05= Strongly Disagree | | [__\|__] |
| 37 | Repeated use of EC pose a health risk  01= Strongly Agree \| 02= Agree \| 03 = Neutral  04= Disagree \| 05= Strongly Disagree | | [__\|__] |
| 38 | EC should be prescribed for a client to have on hand prior to an episode of unprotected sexual intercourse  01= Strongly Agree \| 02= Agree \| 03 = Neutral  04= Disagree \| 05= Strongly Disagree | | [__\|__] |
| 39 | EC should be available without prescription  01= Strongly Agree \| 02= Agree \| 03 = Neutral  04= Disagree \| 05= Strongly Disagree | | [__\|__] |
| 40 | EC should be easily made accessible to all females  01= Strongly Agree \| 02= Agree \| 03 = Neutral  04= Disagree \| 05= Strongly Disagree | | [__\|__] |
| 41 | EC should be used regularly to prevent unwanted pregnancy  01= Strongly Agree \| 02= Agree \| 03 = Neutral  04= Disagree \| 05= Strongly Disagree | | [__\|__] |
| 42 | EC is a safe method of preventing unplanned pregnancy  01= Strongly Agree \| 02= Agree \| 03 = Neutral  04= Disagree \| 05= Strongly Disagree | | [__\|__] |
| 43 | Will use EC in the future when the need arises  01= Strongly Agree \| 02= Agree \| 03 = Neutral  04= Disagree \| 05= Strongly Disagree | | [__\|__] |
| 44 | Will advise family members and friends to use EC  01= Strongly Agree \| 02= Agree \| 03 = Neutral  04= Disagree \| 05= Strongly Disagree | | [__\|__] |
| **Section F: Knowledge on Relationship between Emergency Contraception and Sexually Transmitted Diseases (STI)** | | | |
| 45 | Have you ever heard of Sexually Transmitted Infections (STIs)?  01= Yes \| 02= No | | [__\|__] |
| 46 | If yes, to (Q39), from what source did you gain knowledge about STIs? (tick all that apply)  01= Health care centers  02= Family  03= Friends  04= School  05= Internet  06= TV / Radio  07= Other(s) Specify …………… | | [__\|__]  [__\|__]  [__\|__]  [__\|__]  [__\|__]  [__\|__]  [__\|__] |
| 47 | Have you ever been diagnosed with an STI?  01= Yes \| 02= No | | [__\|__] |
| 48 | If yes, which sexually transmitted infection were you diagnosed with? (**tick all that apply**)  01= HIV/AIDS  02= Gonorrhea  03= Syphilis  04= Genital Wart  05= Human Papillomavirus  06= Herpes  07= Chlamydia  08= Other, Specify ………………. | | [__\|__]  [__\|__]  [__\|__]  [__\|__]  [__\|__]  [__\|__]  [__\|__]  [__\|__] |
| 49 | What kind of regular modern contraception can protect against STIs? (**Tick all that apply)**  01= Injectable Depo provera  02= Implants (e.g. Jadelle)  03= Oral contraceptive pill (e.g. Secure)  04= Mini pills  05= Condom  07= Other, Specify…………………. | | [__\|__]  [__\|__]  [__\|__]  [__\|__]  [__\|__]  [__\|__]  [__\|__]  [__\|__] |
| 50 | Can emergency contraception prevent STIs?  01 = Yes \| 02 = No | | [__\|__] |
| 51 | If yes, what kind of emergency contraception can prevent STIs? (tick all that apply)  01= Combined oral contraception  02= Progesterone only pill. (Eg Postinor 2, Lydia)  03= Intrauterine copper device  04= Ulipristal acetate  99= Don’t know | | [__\|__]  [__\|__]  [__\|__]  [__\|__]  [__\|__] |
| **THANK YOU FOR YOUR TIME** | | | |
