# Supplementary material for: Factors influencing the use of emergency contraceptives among reproductive age women in the Kwadaso Municipality, Ghana
Source: PLoS One. 2022 Mar 3;17(3):e0264619. doi: 10.1371/journal.pone.0264619 (PMC8893659; doi:10.1371/journal.pone.0264619)
Supplement: S2 File — (DOCX) [file pone.0264619.s003.docx]

**APPENDIX II: PARTICIPANT CONSENT FORM**

**CONSENT FORM**

STUDY TITLE: Factors Influencing the Use of Emergency Contraception among Reproductive Age Women in the Kwadaso Municipality, Ghana

PARTICIPANTS’ STATEMENT

I acknowledge that I have read or have had the purpose and contents of the Participants’ Information Sheet read and all questions satisfactorily explained to me in a language I understand. I fully understand the contents and any potential implications as well as my right to change my mind (i.e. withdraw from the research) even after I have signed this form.

I voluntarily agree to be part of this research.

Name of Participant…………………………..

Participants’ Signature ……………………...OR Thumb Print……………………………

Date:………………………………….

INTERPRETERS’ STATEMENT

I interpreted the purpose and contents of the Participants’ Information Sheet to the afore named participant to the best of my ability in the local language to his proper understanding.

All questions, appropriate clarifications sort by the participant and answers were also duly interpreted to his/her satisfaction.

Name of Interpreter……………………………

Signature of Interpreter……………………….. OR Thumb Print ………............................

Date:………………………

Contact Details

STATEMENT OF WITNESS

I was present when the purpose and contents of the Participant Information Sheet was read and explained satisfactorily to the participant in the language, he/she understood

I confirm that he/she was given the opportunity to ask questions/seek clarifications and same were duly answered to his/her satisfaction before voluntarily agreeing to be part of the research.

Name:…………………………

Signature…………………………... OR Thumb Print ………............................

Date:……………………………

INVESTIGATOR STATEMENT AND SIGNATURE

*Brief statement or declaration that investigator has given enough information to participants to make informed decisions.*

I certify that the participant has been given ample time to read and learn about the study. All questions and clarifications raised by the participant have been addressed.

Researcher’s name: ……………………………….

Signature ……………………………

Date: ………………………………….
