# Supplementary material for: Factors influencing the use of emergency contraceptives among reproductive age women in the Kwadaso Municipality, Ghana
Source: PLoS One. 2022 Mar 3;17(3):e0264619. doi: 10.1371/journal.pone.0264619 (PMC8893659; doi:10.1371/journal.pone.0264619)
Supplement: Attachment — Submitted filename: Response to Reviewers.docx [file pone.0264619.s005.docx]

Dear Editors,

We, authors of the manuscript “Factors Influencing Emergency Contraception among Women of Reproductive Age in Kwadaso Municipality, Ghana”, thank the reviewers for their generous comments and have edited the manuscript to address their concerns.

Also, the style of the manuscript has been changed to meet PLOS ONE’s requirements, including those for file naming. All supporting documents noted in the manuscript have been duly provided.

We believe that the manuscript is now suitable for publication in your reputable journal.

Thank you.

Yours sincerely,

Maxwell Afranie Appiah

(Corresponding author)

**RESPONSE TO ACADEMIC EDITOR AND REVIEWERS**

**RESPONSE TO ACADEMIC EDITORS**

Comment 1: Please ensure that your manuscript meets PLOS ONE’s style requirements, including those for file naming.

Response: Manuscript has been updated to meet the PLOS ONE’s style requirements.

Comment 2: You indicated that you had ethical approval for your study. In your methods section, please ensure you have also stated whether you obtained consent from parents or guardians of the minors (<18) included in the study or whether the research ethics committee or IRB specifically waived the need for their consent.

Response: Statement regarding consent obtained from parents or guardians of the minors (<18) has been clarified on page 5 (line 191-192) of the revised manuscript.

Comment 3: Please include additional information regarding the survey or questionnaire used in the study and ensure that you provided sufficient details that others could replicate the analyses.

Response: Additional information regarding the questionnaire has been provided as a supporting information and indicated on page 18 on the revised manuscript.

Comment 4: Please state whether you validated the questionnaire prior to testing on study participants. Please provide details regarding the validated group within the methods section

Response: Details regarding questionnaire validation and validated group has been stated within the methods section on page 4 (147 -152) of the revised manuscript.

Comment 5: Please note that in order to use the direct billing option the corresponding author must be affiliated with the chosen institute. Please either amend your manuscript to change the affiliation or corresponding author, or email us at [plosone@plos.org](mailto:plosone@plos.org) with a request to remove this option.

Response: Authors applied for the PLOS Global Participation Initiative (GPI) funding at time of manuscript submission.

**RESPONSE TO REVIEWER 1**

**Abstract**

Comment: Kindly provide the key points of your methodology as the current text is hard to follow

Response: The problem with methodology in the abstract has been clarified on page 1 of the revised manuscript.

**Introduction**

Comment: Authors should write precisely why they are conducting this study? A lot of published studies are available on this topic, kindly mention clearly what the current study will add.

Response: The purpose for conducting the study and its significants have been reported on page 3 (line 101 – 108) of the revised manuscript

**Methods**

Comment: The authors did not provide references to existing literature from which they adopted the survey tool.

Response: Reference to existing literature from which questionnaire was adopted as been reported on page 4 (line 146) of the revised manuscript.

Comment: Authors should discuss the questionnaire under a separate heading and should also provide it as a supplementary file.

Response: Questionnaire has been discussed under a separate heading. The questionnaire has been included as a supporting document in the manuscript.

Comment: Authors should also provide details about their scoring criteria and on what basis they categorized knowledge and attitude of the study participants.

Response: Details about the scoring criteria has been reported on page 5 (line 165 -178) of the revised manuscript.

**Results**

Comment: The authors did not provide detail on which part of the variable (YES or No) they applied the logistic regression analysis.

Response: Detail on the variable logistic regression analysis was applied has been reported on page 5 (line 178-181) of the revised manuscript.

Comment: The authors should also provide detail about references used in the logistic regression analysis. If “1” indicate the reference value then how there could be two reference values from one variable that is presented in table 5?

Response: Detail about reference used in logistic regression analysis has been reported. The reference value was represented with “1”, and the two reference values from one variable has been corrected.

**Discussion**

Comment: The author should discuss the methodological limitations and strengths of the study more precisely.

Response: Methodological limitations and strength have been precisely discussed.

**Conclusion**

Comment: A more logical conclusion should be provided based on the results with significant importance and contribution of the study tp the scientific literature. The results should not be repeated in conclusion

Response: Problem with conclusion has been clarified page 15 and 16 of the revised manuscript

**RESPONSE TO REVIEWER 2**

Comment 1: The are a number of typos and grammatical errors throughout the manuscript.

Response: All grammatical errors have been corrected.

Comment 2: Authors are to provide reference from where they have adopted the questions for the questionnaire.

Response: Reference to existing literature from which questionnaire was adopted as been reported on page 4 (line 146) of the revised manuscript.

Comment 3: Explain the process of questionnaire translation into the local language, and how it reliability and validity was examined?

Response: The process of questionnaire translation into local language has been reported on page 4 of the revised manuscript.

Comment 4: Besides nausea and vomiting there are many other clinically significant untoward effects associated with ECPs, eg. Menstrual irregularity, breast tenderness and ectopic pregnancy. Authors are required to discuss these as well in the introduction section of their manuscript, and discuss how these could influence ECPs use.

Response: Other clinically significant untoward effects associated with ECPs have been reported in the introduction section as required.

Comment 5: As ECPs cannot prevent the chances of sexually transmitted diseases how could this aspect affect the person’s attitude towards ECP use? Secondly, the cost of ECPs must be explored for its use.

Response: The cost of ECPs has been reported on page 1 (line 68 – 69) of the revised manuscript. The study did not explore the relationship between EC use and sexually transmitted diseases. Sexually transmitted diseases were only captured as part of the knowledge assessment questions on ECs. We hope to explore the how STIs affects attitude towards EC use in future research.
