# Supplementary material for: Factors influencing the use of emergency contraceptives among reproductive age women in the Kwadaso Municipality, Ghana
Source: PLoS One. 2022 Mar 3;17(3):e0264619. doi: 10.1371/journal.pone.0264619 (PMC8893659; doi:10.1371/journal.pone.0264619)
Supplement: Attachment — Submitted filename: Review.docx [file pone.0264619.s006.docx]

**Review and Comments**

Comment 1: Authors need to limit the use of similar sentences through the manuscript.

Comment 2: Under the heading of introduction, it is mentioned that several studies have been conducted in Ghana regarding the factors influencing the use of EC in female college or university students. Authors need to add reference here.

Comment 3: Sampling or selection of one married women out of more than one in a selected household is done by simple random sampling. Clarify if it is random or convenient sampling as no explanation is given in this context?

Comment 4: In ‘Data Analysis’ section, authors have used 50% score as cut off value to describe knowledge as ‘poor’ or ‘good’ and attitude as ‘negative’ or ‘positive’. Provide the basis or reference for this interpretation.

Comment 5: It would be better if authors add a tabulated format under the heading of introduction describing the commonly used EC brands and their prices in Ghana instead of summing up in one sentence.
