# Supplementary material for: Factors influencing the use of emergency contraceptives among reproductive age women in the Kwadaso Municipality, Ghana
Source: PLoS One. 2022 Mar 3;17(3):e0264619. doi: 10.1371/journal.pone.0264619 (PMC8893659; doi:10.1371/journal.pone.0264619)
Supplement: Attachment — Submitted filename: Response to Reviewers.docx [file pone.0264619.s007.docx]

Dear Editor,

We, authors of the manuscript “Factors Influencing the Use of Emergency Contraceptives among Women of Reproductive Age in Kwadaso Municipality, Ghana”, thank the reviewers for their generous comments and have edited the manuscript to address their concerns.

We believe that the manuscript is now suitable for publication in your reputable journal.

Thank you.

Yours sincerely,

Maxwell Afranie Appiah

(Corresponding author)

**RESPONSE TO REVIEWER #3’s REVIEWS AND COMMENTS**

Comment 1: Authors need to limit the use of similar sentences through the manuscript.

Response: The manuscript has been read thoroughly to remove similar sentences.

Comment 2: Under the heading of introduction, it is mentioned that several studies have been conducted in Ghana regarding the factors influencing the use of EC in female college or university students. Authors need to add reference here.

Response: References to the above statement have been added and reported on page 3 (line 100) of the revised manuscript

Comment 3: Sampling selection of one married woman out of more than one in a selected household is done by simple random sampling. Clarify if it is random or convenient sampling as no explanation is given in this context?

Response: Explanation to the selection of one woman from many in a household has been well clarified and been reported on page 4 (line 139-143) of the revised manuscript.

Comment 4: In ‘Data Analysis’ section, authors have used 50% score as cut off value to describe knowledge as “poor” or “good” and attitude as ‘negative’ or ‘positive’. Provide the basis or reference for this interpretation.

Response: Authors have revised the cut off point for scoring knowledge into ‘poor’ or ‘good’ and attitude into ‘negative’ or ‘positive’. This has been reported on page 5 (line 174 to 195).

Authors have used **the mean score cut off point** to dichotomize knowledge and attitude level, and have given a thorough interpretation as well as provided reference to this effect. After this revision, all sections of the manuscript which were affected have been updated including the results, discussion, and abstract.

Comment 5: It would be better if authors add a tabulated format under the heading of introduction describing the commonly used EC brands and their prices in Ghana instead of summing up in one sentence.

Response: Authors acknowledge the reviewers concern regarding adding a tabulated format of EC brand and prices to the heading of the introduction. However, authors feel it will be more convenient to maintain the price ranges indicated in the manuscript for reasons that authors may not be able to provide reference for each price of EC brand. Also, most pharmacies in Ghana do not have price specific for EC brands due to the dollar rate instability.

Thank you.
